# Exosomal transfer of functional small RNAs mediates cancer‐stroma communication in human endometrium

**DOI:** 10.1002/cam4.545

**Published:** 2015-12-24

**Authors:** Yoshiko Maida, Masahiro Takakura, Takumi Nishiuchi, Tanihiro Yoshimoto, Satoru Kyo

**Affiliations:** ^1^ Department of Obstetrics and GynecologyKanazawa University School of MedicineKanazawaJapan; ^2^ Department of Molecular PharmacologyKanazawa University School of MedicineKanazawaJapan; ^3^ Institute for Gene ResearchKanazawa University Advanced Science Research CenterKanazawaJapan; ^4^ Department of Obstetrics and GynecologyShimane University School of MedicineIzumoJapan

**Keywords:** Cancer, endometrium, exosome, fibroblast, microRNA

## Abstract

Exosomes are small membrane vesicles secreted from a variety of cell types. Recent evidence indicates that human cells communicate with each other by exchanging exosomes. Cancer cells closely interact with neighboring stromal cells, and together they cooperatively promote disease via bidirectional communication. Here, we investigated whether exosomes can play roles in intercellular communication between cancer cells and neighboring fibroblasts. Endometrial fibroblasts were isolated from normal endometrial tissues and from endometrial cancer tissues, and cell‐to‐cell transfer of endometrial cancer cell line Ishikawa‐derived exosomes was examined. The isolated fibroblasts were cultured in conditioned media from CD63‐GFP‐expressing Ishikawa cells, and we found that GFP‐positive exosomes were transferred from Ishikawa cells to the fibroblasts. Next, we introduced a shRNA for a luciferase gene into Ishikawa cells. This shRNA was encapsulated into exosomes, was transferred to the fibroblasts, and then downregulated luciferase expression in the fibroblasts. The mature microRNAs naturally expressed in Ishikawa‐derived exosomes were also transported into the endometrial fibroblasts, and they altered the microRNA expression profiles of the fibroblasts. These results indicated that endometrial cancer cells could transmit small regulatory RNAs to endometrial fibroblasts via exosomes. Our findings document a previously unknown mode of intercellular communication between cancer cells and related fibroblasts in human endometrium.

## Introduction

Progression of carcinoma is not solely achieved by malignant epithelial cells; the surrounding tumor stroma is also involved in advance of the disease 1, 2. Fibroblasts are the most abundant cell type in tumor stroma. They alter extracellular matrix composition, promote angiogenesis, and mediate the inflammatory responses. These effects contribute to progression and even initiation of carcinomas 2, 3, 4. Although the majority of reported tumor‐stroma interactions are mediated by secreted soluble factors 2, 3, 4, emerging evidence indicates that there is another type of tumor‐stroma communication mediated by secreted vesicles 5, 6, 7.

Exosomes are extracellular membrane vesicles secreted from almost all human cell types 8, 9. They are formed through inward budding of endosomal membranes; this results in the accumulation of intraluminal vesicles within multivesicular bodies (MVBs). MVBs fuse with the plasma membrane and release exosomes into the extracellular milieu. Exosomes contain various molecules, and accumulating evidence indicates that exosomal cargo is transferred from donor cells to recipient cells and plays roles in cell‐to‐cell communication 5, 6, 9, 10, 11. Cancer‐derived exosomes are believed to modify tumor biology via interactions with both cancer cells and stromal cells 12, 13, 14, 15, 16, 17. Fibroblasts almost certainly receive exosomal molecules from cancer cells, however, little is known about exosome‐mediated cancer‐to‐fibroblast communication 18, 19.

Here, we examined whether cancer‐derived exosomes transport exosomal cargo, especially functional small RNAs, to neighboring fibroblasts. Our data clearly demonstrated that the exosomes secreted from endometrial cancer cells were accepted by endometrial fibroblasts and that these exosomes transported functional small RNA cargo to the fibroblasts. This report is the first to demonstrate that endometrial cancer cells communicate with neighboring fibroblasts by transferring small regulatory RNA via exosomes.

## Materials and Methods

### Isolation of human endometrial fibroblasts

Normal endometrial tissues and endometrial cancer tissues were obtained from patients undergoing hysterectomy as treatment with written informed consent. Endometrial fibroblasts were isolated as reported previously 20. The use of clinical materials was approved by the Institutional Research Ethics Committee.

### Immunocytochemistry

Immunocytochemistry was performed as described previously 21. The following antibodies were used: anti‐pan‐Cytokeratin (4/5/6/8/10/13/18) (C11; Santa Cruz Biotechnology, Santa Cruz, CA) and anti‐CD10 (FR4D11; Santa Cruz Biotechnology).

### Plasmids

pCT‐CD63‐GFP was purchased from System Biosciences (Mountain View, CA). MISSION Non‐Target shRNA Control Vector and MISSION Luciferase shRNA Control Vector were purchased from Sigma‐Aldrich Japan (Tokyo, Japan). pGL3‐Control Vector was purchased from Promega (Madison, WI). The sensor vector for luc shRNA was constructed by introducing a binding site with perfect complementarity to luc shRNA into *Xba*I site of psiCHECK‐2 Vector (Promega). The sequences of the binding site are as follows: 5′‐CGCTGAGTACTTCGAAATGTC‐3′ (sense) and 5′‐GACATTTCGAAGTACTCAGCG‐3′ (antisense). Sensor vectors for hsa‐miR‐141‐3p and hsa‐miR‐200b‐3p were constructed by introducing three tandem binding sites with perfect complementarity to each microRNA (miRNA) into *Xho*I and *Not*I sites of psiCHECK‐2 Vector. The sequences of the binding sites are as follows: 5′‐CCATCTTTACCAGACAGTGTTA‐3′ (sense) and 5′‐TAACACTGTCTGGTAAAGATGG‐3′ (antisense) for hsa‐miR‐141‐3p, 5′‐TCATCATTACCAGGCAGTATTA‐3′ (sense) and 5′‐TAATACTGCCTGGTAATGATGA‐3′ (antisense) for hsa‐miR‐200b‐3p.

### Cell culture and stable expression of CD63‐GFP and shRNAs

Human endometrial cancer cell line Ishikawa and endometrial fibroblasts were cultured in Dulbecco's Modified Eagle's medium (DMEM) containing 10% heat‐inactivated fetal bovine serum (FBS) at 37°C in humidified CO_2_ incubator.

For labeling of exosomes, pCT‐CD63‐GFP was packaged into pseudotyped lentiviruses using pPACKH1 Lentivector Packaging Kit (System Biosciences) and delivered to Ishikawa cells according to the manufacturer's protocol. MISSION Non‐Target shRNA Control Vector or MISSION Luciferase shRNA Control Vector was packaged into amphotropic lentiviruses using MISSION Lentiviral Packaging Mix (Sigma‐Aldrich Japan) and delivered to Ishikawa cells according to the manufacturer's protocol.

### Preparation of conditioned medium and isolation of exosomes

To prepare the conditioned medium containing fluorescently labeled exosomes, the culture medium of CD63‐copGFP‐expressing Ishikawa cells was passed through a 0.20 *μ*m filter.

Prior to exosome isolation, Ishikawa cells were starved of serum for 2 days. After incubation, the culture medium was collected and centrifuged at 500*g* for 10 min and at 16,500*g* for 20 min at room temperature; the medium was then passed through a 0.20 *μ*m filter. Exosomes were pelleted by ultracentrifugation at 110,000*g* for 90 min at 4°C. The pellet was washed twice and resuspended with phosphate‐buffered saline (PBS). In the treatment with the isolated exosomes, the resuspended exosomes were added to the normal growth media to the final concentration of 150 *μ*g/mL.

### Electron microscopy

The resuspended exosomes were dropped on carbon‐filmed grids and negatively stained with 2% uranyl acetate. The exosomes were observed with a transmission electron microscope (JEOL JEM‐2000EX, Tokyo, Japan).

### Flow cytometry

Cell cultures were maintained in either conventional growth media or the conditioned media prepared from CD63‐copGFP‐expressing Ishikawa cells for 10 days. The medium was renewed every 24 h. After the 10‐day incubation, the cells were analyzed with a JSAN desktop cell sorter (Bay bioscience, Kobe, Japan) using AppSan software (Bay bioscience).

### Immunoblotting

Exosomes were lysed in RIPA buffer (Cell Signaling, Danvers, MA). Extracted proteins were separated on sodiumdodecyl sulfate–polyacrylamide gel and transferred to a polyvinylidene difluoride (PVDF) membrane. The membrane was blocked, incubated with anti‐human CD63 antibody (MX‐49.129.5; Santa Cruz Biotechnology), and then with horseradish peroxidase‐linked secondary antibody (NA931V; GE Healthcare, Buckinghamshire, UK). The membrane was then subjected to enhanced chemiluminescence.

### RNA isolation

Total RNAs or small RNA fractions of <200 nucleotides were extracted with miRNeasy Mini Kit (Qiagen, Hilden, Germany). Total RNAs for quantification of primary miRNAs (pri‐miRNAs) were treated with RNase‐Free DNase Set (Qiagen). The Agilent 2100 Bioanalyzer (Agilent Technologies, Santa Clara, CA) for total RNA (RNA pico chips) and for small RNA (small RNA chips) were used to assess the profiles of RNAs.

### Quantitative real‐time PCR

To quantify pri‐miRNA and mRNA, 1 *μ*g of total RNA was reverse transcribed using SuperScript First‐Strand Synthesis System (Invitrogen, Carlsbad, CA). The quantification of mature miRNA, pri‐miRNA, and mRNA was performed by TaqMan real‐time PCR assays (Applied Biosystems, Foster City, CA) according to manufacturer's instruction. The following TaqMan miRNA Assays, TaqMan Pri‐miRNA Assays, and TaqMan Gene Expression Assay were used: hsa‐miR‐141‐3p (000463), hsa‐miR‐200b‐3p (002251), RNU6B (001093), pri‐miRNA of hsa‐miR‐141 (Hs03303157_pri), pri‐miRNA of hsa‐miR‐200b (Hs03303027_pri), GAPDH (Hs99999905_m1). *RNU6B* and *GAPDH* were used as references. To measure the levels of the guide strand (5′‐GACATTTCGAAGTACTCAGCG‐3′) of MISSION Luciferase shRNA, Custom TaqMan Small RNA Assay was designed and purchased from Applied Biosystems. Real‐time PCR was performed in triplicate with ABI PRISM 7000 Sequence Detection System (Applied Biosystems).

### Luciferase assay

Cells were cultured in 24‐well dishes and transfected with 0.1 *μ*g of luciferase reporter plasmids using FuGENE HD Transfection Reagent (Promega) according to the manufacturer's protocol. In experiments with and without exosome treatment, the treatment was started at 6 h after the transfection. The luciferase activity was assessed 48 h after transfection in triplicate with Dual‐Luciferase Reporter Assay System (Promega).

### Microarray analysis

miRNA microarray analysis was performed basically according to the Agilent miRNA Microarray System Protocol (Agilent Technologies). Total RNA (100 ng) was used to prepare 3′ end‐labeled miRNA with Cy3‐pCp using the miRNA Complete Labelling Reagent and Kit (Agilent Technologies). Hybridization solutions containing Cy3‐labeled miRNA were applied to the Agilent human miRNA Microarray 8 X 15k Rel.15.0 (Agilent Technologies). Then, an Agilent microarray scanner (G2565BA; Agilent Technologies) was used to scan the arrays with the maximum laser intensity in the Cy3 channel. Feature Extraction Software (Ver. 10.7.3.1; Agilent Technologies) was then used to analyze the images. These data were analyzed further with GeneSpring GX11 software (Agilent Technologies). If necessary, normalization was performed by using per‐chip 90th‐percentile method.

### Statistics

Unpaired *t*‐tests were performed using Statcel3 software (OMS publishing, Saitama, Japan). *P *<* *0.05 was considered significant.

## Results

**Figure 1:**
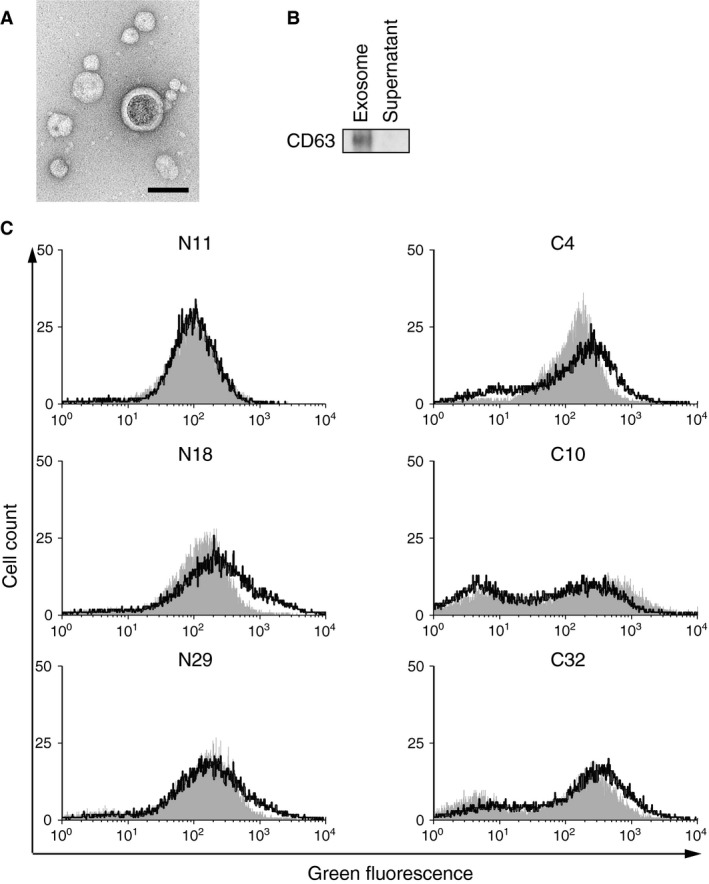
Intercellular transfer of Ishikawa‐derived exosomes to endometrial fibroblasts. (A) Transmission electron microscopy image of Ishikawa‐derived exosomes. The scale bar indicates 100 nm. (B) Ishikawa‐derived exosomes and the supernatant after ultracentrifugation were analyzed on immunoblots stained with an anti‐CD63 antibody. (C) Flow cytometry of endometrial fibroblasts. The cells were treated with either the conditioned media (black) prepared from CD63‐copGFP‐expressing Ishikawa cells or with the conventional media (gray).

To determine exosome secretion from human endometrial cancer Ishikawa cells, medium from Ishikawa cultures was subjected to a standard ultracentrifugation and microfiltration protocol to isolate exosomes 10, 12, 22. Transmission electron micrographs revealed that the collected products had a distinctive cup shape and diameters ranging from 30 to 100 nm (Fig. 1A). The isolated products expressed CD63, an exosomal marker (Fig. 1B). The characteristics of the products were compatible with those of exosomes, indicating that exosomes were secreted from Ishikawa cells and that we had successfully isolated Ishikawa‐derived exosomes.

To investigate intercellular transfer of exosomes, Ishikawa‐derived exosomes were labeled with copepod green fluorescent protein (copGFP). A fusion protein comprised of CD63 and copGFP (CD63‐copGFP) was transduced into Ishikawa cells to prepare donor cells that produced copGFP‐labeled exosomes, and the conditioned medium from the cells was used for the subsequent experiments. We first treated Ishikawa cells, which did not carry the CD63‐copGFP transgene, with the conditioned medium because exosome transfer among cells of the same type had been reported 5, 10, 12, 22. After a 10‐day treatment, copGFP signal accumulated to Ishikawa cells treated with the conditioned media (Fig. S1A). The increased copGFP expression in the Ishikawa cells was also confirmed by flow cytometry (Fig. S1B). These data indicated that exosomes were exchanged among Ishikawa cells.

Next, we investigated whether Ishikawa‐derived exosomes could be transferred to endometrial fibroblasts. Normal endometrial fibroblasts and fibroblasts derived from endometrial cancer were prepared from clinical samples (Table 1 and Fig. S2). Each type of fibroblast was treated with either conventional media or conditioned media from CD63‐copGFP‐expressing Ishikawa cells for 10 days, and analyzed by flow cytometry and fluorescent microscopy (Fig. 1C and Fig. S3). Of six endometrial fibroblast lines, four lines, including two normal endometrial fibroblasts (N18 and N29) and two endometrial cancer‐derived fibroblasts (C4 and C32), exhibited apparently elevated expression of green fluorescence after the treatment with the conditioned media; additionally, one normal endometrial fibroblast (N11) showed a slight increase. These findings indicated that copGFP‐expressing exosomes were incorporated into the fibroblasts from the conditioned media. Only an endometrial cancer‐derived fibroblast (C10) exhibited a small decrease in green fluorescence following treatment with the conditioned media. Taken together, these results indicated that exosomes may be transferred from Ishikawa cells to most types of endometrial fibroblasts, both normal and cancer‐derived.

**Table 1:** Characteristics of patients that donated endometrial tissues for fibroblast isolation

Cell no.	Age	Hormonal status	Histological diagnosis
Normal endometrial fibroblast			
N11	44	Proliferative	Leiomyoma
N18	41	Secretory	Leiomyoma
N29	45	Proliferative	Leiomyoma
Endometrial cancer‐derived fibroblast			
C4	57	Postmenopausal	Endometrioid adenocarcinoma, G3
C10	62	Postmenopausal	Mixed adenocarcinoma
C32	41	Proliferative	Endometrioid adenocarcinoma, G1

### Small RNAs were transported from Ishikawa cells to endometrial fibroblasts via exosomes

To determine whether RNAs enclosed within endometrial cancer‐derived exosomes can be incorporated into endometrial fibroblasts, the specific RNA profiles of Ishikawa‐derived exosomes were examined. Total RNA samples extracted from Ishikawa‐derived exosomes and from the parent Ishikawa cells demonstrated completely different distributions of RNA sizes (Fig. S4). The exosome sample was highly enriched with short RNAs (<200 nucleotides long), whereas the cellular sample had peaks representing ribosomal RNAs as reported for other cell types 10, 12, 22. Further analysis revealed that the short RNA fraction from the exosome sample included RNAs that were 20 nucleotides or longer (Fig. S4, right). These findings indicated that small non‐coding RNAs, such as miRNAs, were cargo of Ishikawa‐derived exosomes.

**Figure 2:**
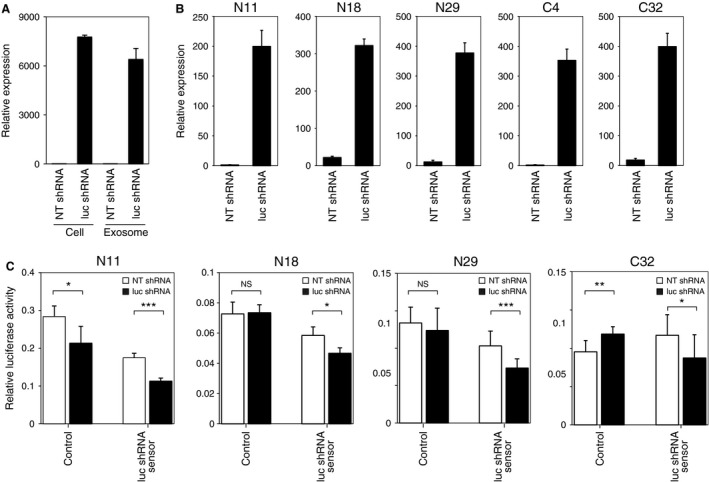
Ishikawa‐derived exosomes transport exogenous shRNA to endometrial fibroblasts. (A) Confirmation of expression of the transduced luc shRNA in Ishikawa cells and Ishikawa‐derived exosomes by qRT‐PCR. Cellular and exosomal total RNAs were extracted from Ishikawa cells stably transduced with non‐target control shRNA (NT shRNA) or luciferase shRNA (luc shRNA), respectively. The RNAs were applied to qRT‐PCR for the guide strand of the luc shRNA. Values represent mean ± SD. (B) qRT‐PCR for luc shRNA. Endometrial fibroblasts were treated with either NT shRNA‐containing (NT shRNA) or luc shRNA‐containing (luc shRNA) Ishikawa‐derived exosomes. Enriched small RNA species were used for the analysis. Values represent mean ± SD. (C) Gene silencing activity of luc shRNA in endometrial fibroblasts. Endometrial fibroblasts were transfected with psiCHECK‐2 Vector (control) or the luc shRNA sensor vector (luc shRNA sensor), and then treated with either NT shRNA‐containing (white bars) or luc shRNA‐containing (black bars) exosomes. Values represent mean ± SD (*P <* *0.05; **P <* *0.01; ***P <* *0.001; NS, not significant).

In order to explicitly test whether functional small RNAs were transferred from cell to cell via exosomes, intercellular transportation of exogenous small RNAs that were not naturally expressed in human cells was examined. A short hairpin RNA (shRNA) that targeted firefly luciferase (luc shRNA) or a non‐target control shRNA (NT shRNA) was stably transduced into Ishikawa cells. The specific and constitutive expression of luc shRNA in the transduced Ishikawa cells and their exosomes was confirmed by quantitative RT‐PCR (qRT‐PCR) (Fig. 2A). Exosomes were isolated from culture media of NT shRNA‐expressing or luc shRNA‐expressing Ishikawa cells and used for the treatment of endometrial fibroblasts. After a 5‐day treatment, luc shRNA was obviously expressed in endometrial fibroblasts treated with the luc shRNA‐containing Ishikawa‐derived exosomes, whereas the expression of luc shRNA was below background levels in the cells treated with NT shRNA‐containing exosomes (Fig. 2B). The results clearly indicated that both normal endometrial fibroblasts and endometrial cancer‐derived fibroblasts were capable of incorporating small RNAs from Ishikawa‐derived exosomes. We then used the luciferase reporter system to evaluate the RNA silencing activity of the incorporated luc shRNA. The luc shRNA targets perfectly complementary sequences on firefly luciferase gene and thereby represses luciferase expression (Fig. S5A). The psiCHECK‐2 Vector contains a modified firefly luciferase (Fig. S5C). To make the vector sensitive to the shRNA, a perfectly complementary sequence to the luc shRNA was inserted into the 3′ UTR of the firefly luciferase of the psiCHECK‐2 Vector (Fig. S5B). The psiCHECK‐2 Vector or the reconstructed luc shRNA sensor vector was transiently transfected into endometrial fibroblasts, and the cells were then treated with exosomes derived from either NT shRNA‐expressing or luc shRNA‐expressing Ishikawa cells (Fig. 2C). Two normal endometrial fibroblasts (N18 and N29) and an endometrial cancer‐derived fibroblast (C32) exhibited significant repression of firefly luciferase activity derived from the luc shRNA sensor vector upon the treatment with the luc shRNA‐containing exosomes. Although treatment with exosomes derived from different donors affected the baseline expression levels of the luciferases in N11 cells, reduction of firefly luciferase expression was more prominent for the luc shRNA sensor vector than for the control psiCHECK‐2 Vector upon the treatment with the luc shRNA‐containing exosomes. Taken together, these results clearly indicated that functional small RNAs were transferred from Ishikawa cells to endometrial fibroblasts.

### Different miRNA expression profiles between endometrial fibroblasts, Ishikawa cells, and Ishikawa‐derived exosomes

**Figure 3:**
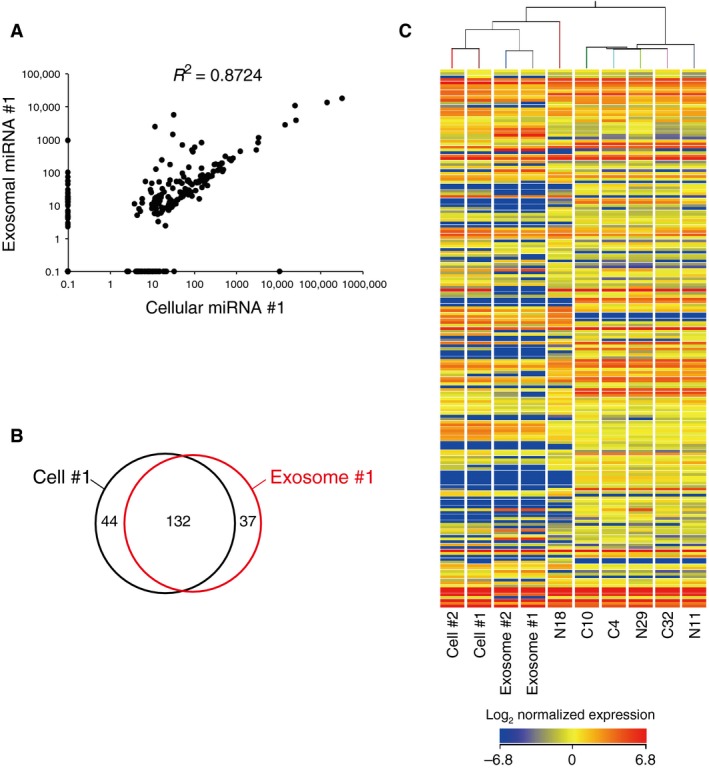
miRNA expression profiles of Ishikawa cells, Ishikawa‐derived exosomes and endometrial fibroblasts. miRNA expression profiles of individual cell types and exosomes were analyzed by miRNA microarray. (A) A scatter plot of miRNA expression levels within Ishikawa cells or Ishikawa‐derived exosomes for one (#1) of the repeated experiments. See also Figure S6. (B) Venn diagram showing the overlap between miRNA signature of Ishikawa cells (Cell #1, black) and that of Ishikawa‐derived exosomes (Exosome #1, red) from Experiment #1. (C) Heat map and cluster analysis of miRNA expression in Ishikawa cells (Cell #1 and #2), Ishikawa‐derived exosomes (Exosome #1 and #2), and endometrial fibroblasts.

Prior to investigating the intercellular transfer of exosomal miRNAs, we performed miRNA microarrays to profile the miRNA expression in each cell type and exosome type used in our study. Approximately 170–200 different miRNAs were found in Ishikawa cells and in Ishikawa‐derived exosomes in the repeated experiments (Fig. 3A and B, Table S1 and Fig. S6). Interestingly, about 75% of the miRNAs found in the cells were also found in the exosomes, but about 22% of miRNAs detected in the exosomes were not found in the cells. Consistent with this, the coefficient of determination between the cellular miRNAs and the exosomal miRNAs was slightly weak (*R* ^2^ = 0.87) (Fig. 3A and Fig. S6A) while Experiment #1 and Experiment #2 demonstrated a strong correlation in both the cellular miRNAs and the exosomal miRNAs (*R* ^2^ = 0.99) (Fig. S6C). The cellular‐to‐exosome expression ratios for individual miRNAs expressed in both cells and exosomes varied substantially from one miRNA to the next; actually, some miRNAs were very abundant in exosomes, but rare in cells, and vice versa, suggesting selective encapsulation of cellular miRNAs into exosomes.

miRNA profiling of endometrial fibroblasts uncovered a distinctive miRNA signature of these cell lines (Fig. 3C and Table S2). In a hierarchical clustering analysis, five of six endometrial fibroblasts were categorized into a single cluster that was distinct from Ishikawa cells and Ishikawa‐derived exosomes. Of those five endometrial fibroblasts, four (N29, C4, C10, and C32) were further classified into the nearest group. The only exception was the normal endometrial fibroblast (N18) that was grouped into the same cluster with Ishikawa cells, suggesting a distinctive character of N18 cells among endometrial fibroblasts.

### Exosomes transport functional miRNAs from Ishikawa cells to endometrial fibroblasts

**Figure 4:**
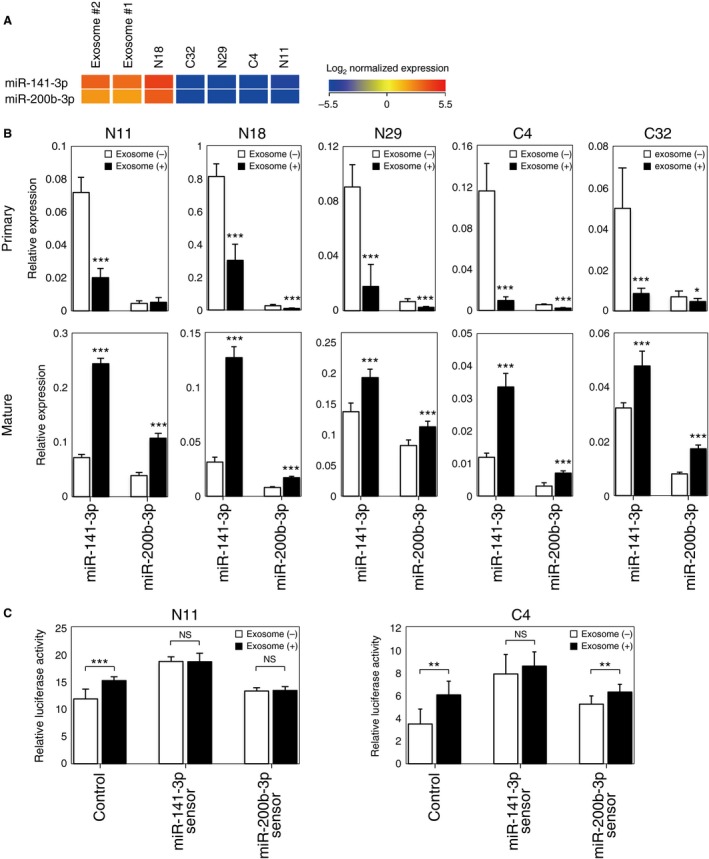
Increase of functional mature miRNAs in endometrial fibroblasts treated with Ishikawa‐derived exosomes. (A) Heat map comparing the selected miRNA expression between Ishikawa‐derived exosomes and endometrial fibroblasts. (B) qRT‐PCR for primary (upper panels) and mature (lower panels) miRNAs. Endometrial fibroblasts were treated with (black bars) or without (white bars) Ishikawa‐derived exosomes. Values represent mean ± SD (*P <* *0.05; ***P <* *0.001). (C) Decreased luciferase activity in endometrial fibroblasts with the miRNA sensor vectors and the exosome treatment. Endometrial fibroblasts were transfected with psiCHECK‐2 Vector (control) or the miRNA sensor vectors (miR‐141‐3p sensor and miR‐200b‐3p sensor), then incubated with (black bars) or without (white bars) Ishikawa‐derived exosomes. Values represent mean ± SD (**P <* *0.01; ***P <* *0.001; NS, not significant).

To investigate whether Ishikawa‐derived exosomes carry endogenous miRNAs to endometrial fibroblasts, we first examined two miRNAs, miR‐141‐3p and miR‐200b‐3p, that were very abundant in Ishikawa‐derived exosomes and almost absent from the fibroblasts (Fig. 4A). Endometrial fibroblasts were treated with either of two types of normal growth media, that with or that without Ishikawa‐derived exosomes. After a 5‐day treatment, RNAs were extracted from both types of target endometrial fibroblasts, and qRT‐PCR was performed. The abundance of both mature miRNAs (mature miR‐141‐3p and mature miR‐200b‐3p) was significantly higher in cells treated with Ishikawa‐derived exosomes than in those not treated with these exosomes (Fig. 4B, lower panels). Surprisingly, the abundance of the primary transcripts for each miRNAs was significantly lower in the fibroblasts treated with the exosomes than in those not treated with exosomes (Fig. 4B, upper panels). The discrepancy between the findings for the primary transcripts (primary miRNAs) versus those for the mature products (mature miRNAs) in the target fibroblasts treated with the exosomes could indicate that the increased abundance of the mature miRNAs was due to the direct transfer of mature miRNAs from the exosomes to the target cells and not due to a increase in the expression of the endogenous gene within the target fibroblasts. We further analyzed the RNA silencing activity of mature miR‐141‐3p and miR‐200b‐3p by luciferase assay (Fig. 4C). Endometrial fibroblasts were transfected with miRNA sensor vectors or with a control vector; transfected cells were then treated with the normal growth media with or without Ishikawa‐derived exosomes. Although the relative luciferase activity was significantly upregulated in both N11 cells and C4 cells with the control vectors upon the treatment with Ishikawa‐derived exosomes, the upregulation was completely absent from cells with the miR‐141‐3p sensor vectors, suggesting that *Renilla* luciferase expression from the sensor vectors was suppressed due to exosome treatment. Similar results were obtained with N11 cells and the miR‐200b‐3p sensor vector. These results indicated the gene regulatory impacts of the increased mature miRNAs in endometrial fibroblasts upon the treatment with Ishikawa‐derived exosomes.

To further elucidate the effects of Ishikawa‐derived exosomes on genome‐wide miRNA expression in endometrial fibroblasts, the effect of each increment of miRNA upon exosome treatment was analyzed based on the microarray data (Table S3). The incremental changes in expression of each miRNA were calculated by subtracting a normalized intensity observed in cells that were not treated with exosomes from the intensity observed in exosome‐treated cells. For N11, C4, and C32 fibroblasts, the mean of increment of the miRNAs expressed in Ishikawa‐derived exosomes was significantly larger than that of the miRNAs not expressed in the exosomes (Table 2 and Fig. S7). Interestingly, miRNA subgroup analyses of the top 20 or the top 50 most abundant exosome‐derived miRNAs revealed that higher miRNA abundance in the exosomes strongly correlated with larger increments for a particular miRNA in the fibroblasts (Table 2). These results strongly indicated the exosomal miRNAs were transferred directly from Ishikawa cells to endometrial fibroblasts via exosomes.

**Table 2:** Increase of miRNA expression in endometrial fibroblasts treated with Ishikawa‐derived exosomes

Cell no.	miRNA subgroup	Increments of expression (mean ± SEM)	*P* value^a^
N11	Expressed in exosomes (*n* = 203)	1.980 ± 0.984	<1 × 10^−3^
	Top 50 most abundant in exosomes	8.068 ± 3.889	<1 × 10^−12^
	Top 20 most abundant in exosomes	15.663 ± 9.217	<1 × 10^−20^
	Not expressed in exosomes (*n* = 648)	0.012 ± 0.009	
N18	Expressed in exosomes (*n* = 203)	−0.284 ± 0.329	0.112
	Top 50 most abundant in exosomes	−0.760 ± 1.301	0.034
	Top 20 most abundant in exosomes	−0.662 ± 3.247	0.233
	Not expressed in exosomes (*n* = 648)	0.013 ± 0.017	
C4	Expressed in exosomes (*n* = 203)	2.424 ± 1.152	<1 × 10^−3^
	Top 50 most abundant in exosomes	9.221 ± 4.519	<1 × 10^−12^
	Top 20 most abundant in exosomes	17.889 ± 10.650	<1 × 10^−20^
	Not expressed in exosomes (*n* = 648)	0.055 ± 0.028	
C32	Expressed in exosomes (*n* = 203)	1.687 ± 0.666	<1 × 10^−4^
	Top 50 most abundant in exosomes	5.476 ± 2.540	<1 × 10^−13^
	Top 20 most abundant in exosomes	10.379 ± 6.006	<1 × 10^−21^
	Not expressed in exosomes (*n* = 648)	0.042 ± 0.016	

a Relative to the miRNA subgroup with miRNAs not expressed in Ishikawa‐derived exosomes.

## Discussion

Cancer cells and the related fibroblasts communicate with each other through secreted signal molecules 2, 3, 4. In this study, we investigated intercellular communication between endometrial cancer cells and endometrial fibroblasts isolated from either normal endometrium or endometrial cancer. Normal endometrial fibroblasts and endometrial cancer‐derived fibroblasts are reported to be different in a hormone (estradiol and progesterone) response 23 and their secretions 23, 24. Here, however, we demonstrated that endometrial cancer cells communicate directly with both types of endometrial fibroblasts via exosomes.

Exosomes contain functional molecules and transfer them to recipient cells through membrane fusion, endocytosis, phagocytosis, and ligand/receptor interactions 25. Two previous studies investigated the contribution of exosomes to the interactions between cancer cells and fibroblasts. Suetsugu et al. traced the fate of cancer‐derived exosomes by fluorescent imaging of the exosomes 18. They prepared GFP‐tagged CD63‐expressing breast cancer cells, co‐cultured the cells with mouse lung tissue cells, and observed green fluorescence in the lung tissue cells. They also injected the breast cancer cells into mice and found green fluorescence in stromal cells in the lung metastasis as well as the primary tumor. Webber et al. reported that exosomes with high levels of TGF‐*β*1 expression, those which were secreted from methothelioma and prostate cancer cells, trigger primary lung fibroblasts to differentiate as myofibroblasts 19. They demonstrated that the TGF‐*β*1 that was bound to the exosome surface interacted with the cell surface TGF‐*β* receptor I and induced differentiation in exactly the same way as does soluble TGF‐*β*. These data offer intriguing clues to the roles of tumor‐derived exosomes in constructing tumor stroma; however, whether fibroblasts internalize exosomal molecules that are packaged and secreted from cancer cells in the same tissue remained unclear. Our data from this study clearly answered this question; cancer‐derived exosomes do transport their gene regulatory cargo directly to the neighboring fibroblasts.

We observed a wide variety of miRNAs in Ishikawa‐derived exosomes, and some such miRNAs seemed to be selectively enriched in these vesicles. Although the sorting system(s) underlying selective loading of exosomal miRNAs remain unknown, Gibbings et al. found that MVBs are sites of miRNA‐loaded RNA‐inducing silencing complex accumulation. They speculated that crosstalk or direct regulation between miRNA processes and MVB‐regulated pathways, such as intercellular transport, does occur 26.

The microarray analysis summarized in Table 2 demonstrated that the miRNA species that were highly abundant in Ishikawa‐derived exosomes apparently increased in the recipient fibroblasts upon the exosome treatment. We further analyzed the incorporation of two specific miRNAs, miR‐141‐3p, and miR‐200b‐3p (Fig. 4). We observed a significant increase in the abundance of these mature miRNAs and an unexpectedly significant decrease in the abundance of the respective primary transcripts in the endometrial fibroblasts that were treated with Ishikawa‐derived exosomes. miR‐141 and miR‐200b are members of the miR‐200 family. They are expressed mainly in epithelial cells, and they contribute to the suppression of the epithelial‐mesenchymal transition 27, 28. An increased abundance of miR‐200 family members following exosome treatment might have been an unnatural state for the recipient fibroblasts, and accordingly, these recipient cells may have repressed production of the endogenous miRNAs in response to this particular alteration.

As shown in Figure 1C, most lines of endometrial fibroblasts prepared in this study were able to incorporate GFP‐expressing Ishikawa‐derived exosomes, but an endometrial cancer‐derived fibroblast (C10) was not. The C10 fibroblast was isolated from a mixed adenocarcinoma composed of endometrioid adenocarcinoma and clear‐cell adenocarcinoma, and we did not know which component(s) of the cancer sample gave rise to the C10 fibroblasts. The C10 cultures were unable to incorporate the exosomes because these cultures may have originated from different histologic types of endometrial carcinoma compared to those of other fibroblast cultures. N18 fibroblasts exhibited a different and unique characteristic. These cells accepted GFP‐labeled Ishikawa‐derived exosomes, and incorporated the shRNAs and the selected miRNAs present in Ishikawa‐derived exosomes. However, these cells did not show a remarkable increase in the mean of increment of miRNA expression with regard to miRNAs expressed in the exosomes upon the exosome treatment. Some of the miRNAs found in the exosomes actually increased in N18 cells, but a larger number of miRNAs decreased in abundance in these cells following exosome treatment (Fig. S7). On the basis of the unique miRNA expression profile of the N18 fibroblasts, we speculated that N18 cells may incorporate miRNAs from Ishikawa‐derived exosomes as do other fibroblasts, but that these cells dealt with the exogenous miRNAs in a very different manner that did not lead to a remarkable change in the mean of increment of the miRNA expression.

In summary, our findings indicated that some cell‐to‐cell interactions between endometrial cancer cells and endometrial fibroblasts occurred via exosome transfer. The functional miRNAs carried by Ishikawa‐derived exosomes were ultimately incorporated into the fibroblasts, and these miRNAs altered the miRNA expression profiles of the recipient fibroblasts. Our results demonstrated that a previously undocumented mode of intercellular communication between cancer cells and related fibroblasts occurs in endometrium; moreover, our results raise the possibility that cancer cells may modify their surrounding stroma via transfer of exosomal RNAs.

## Conflict of Interest

None declared.

## Supporting information


**Table S1.** miRNA microarray analysis of Ishikawa cells and Ishikawa‐derived exosomes.

**Table S2.** miRNA microarray analysis of endometrial fibroblasts.

**Table S3.** miRNA microarray analysis of endometrial fibroblasts treated with or without Ishikawa‐derived exosomes.

**Figure S1.** Exosome transfer among Ishikawa cells.**Figure S2.** Immunocytochemical analysis of endometrial fibroblast.**Figure S3.** Endometrial fibroblasts incorporate Ishikawa‐derived exosomes.**Figure S4.** Characterization of exosomal RNAs.**Figure S5.** luc shRNA targets perfectly complementary sequences.**Figure S6.** Correlation of miRNA expression profiles observed in Ishikawa cells and Ishikawa‐derived exosomes.**Figure S7.** Increased expression of the exosomal miRNAs in endometrial fibroblasts treated with Ishikawa‐derived exosomes.
