# Supplementary material for: Exosomal transfer of functional small RNAs mediates cancer‐stroma communication in human endometrium
Source: Cancer Med. 2015 Dec 24;5(2):304–14. doi: 10.1002/cam4.545 (PMC4735775; doi:10.1002/cam4.545)
Supplement: Supplementary file 4 — Figure S1. Exosome transfer among Ishikawa cells.Figure S2. Immunocytochemical analysis of endometrial fibroblast.Figure S3. Endometrial fibroblasts incorporate Ishikawa‐derived exosomes.Figure S4. Characterization of exosomal RNAs.Figure S5. luc shRNA targets perfectly complementary sequences.Figure S6. Correlation of miRNA expression profiles observed in Ishikawa cells and Ishikawa‐derived exosomes.Figure S7. Increased expression of the exosomal miRNAs in endometrial fibroblasts treated with Ishikawa‐derived exosomes. [file CAM4-5-304-s004.doc]

**Supporting Figure S1_Maida**

**
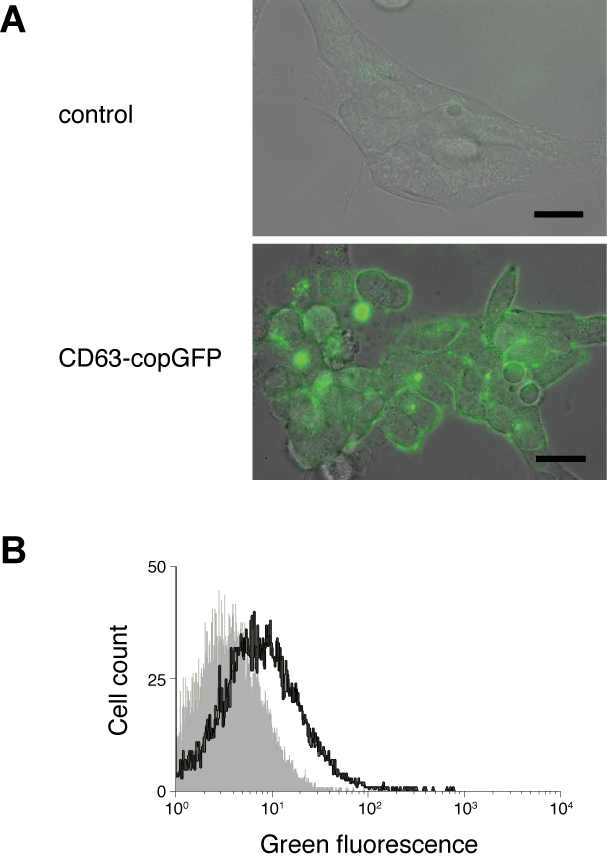
**

**Supporting Figure S1. Exosome transfer among Ishikawa cells.**

Ishikawa cells were treated either with conventional media (control) or with conditioned media prepared from CD63-copGFP-expressing Ishikawa cells (CD63-copGFP) for 10 days. Accumulation of green fluorescence was observed in the cells treated with CD63-copGFP.  **A.** Fluorescence microscope images of the Ishikawa cells after treatment. Scale bars indicate 20 μm. **B.** Flow cytometry of Ishikawa cells treated with control (gray) or CD63-copGFP (black) media.

**Supporting Figure S2_Maida**


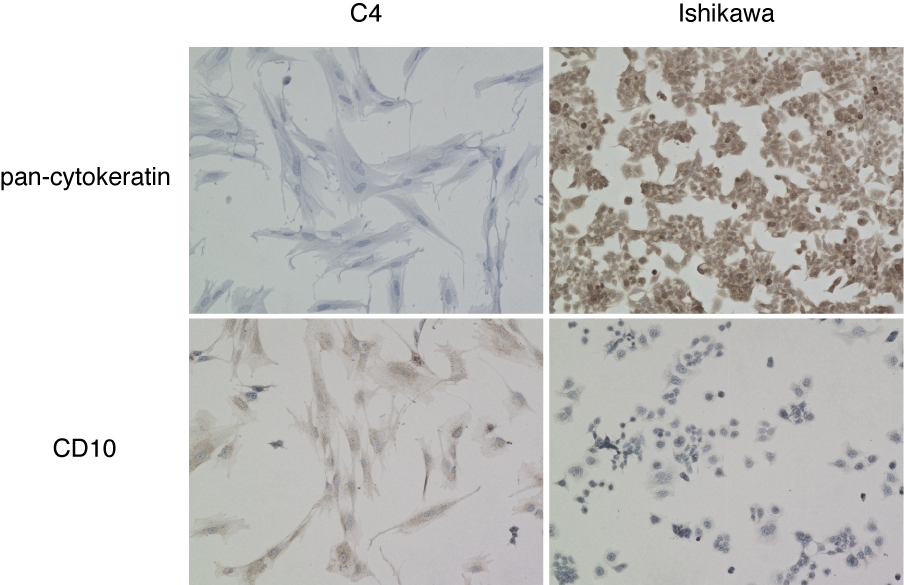


**Supporting Figure S2. Immunocytochemical analysis of endometrial fibroblast.**

To verify stromal origin, newly prepared endometrial fibroblasts were examined by immunocytochemistry. Positive staining for CD10 and negative staining for cytokeratine indicated that the prepared fibroblasts were of stromal origin. Ishikawa cells were used as control for epithelial cells.

**Supporting Figure S3_Maida**


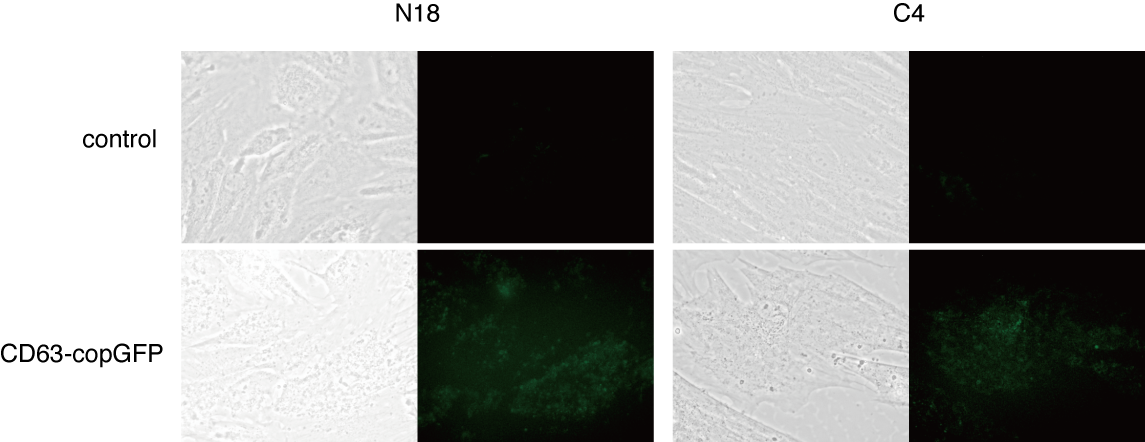


**Supporting Figure S3. Endometrial fibroblasts incorporate Ishikawa-derived exosomes.**

Fluorescence microscope images of a normal endometrial fibroblast (N18) and an endometrial cancer-derived fibroblast (C4). The endometrial fibroblasts were treated either with conventional media (control) or with conditioned media prepared from CD63-copGFP-expressing Ishikawa cells (CD63-copGFP) for 10 days.

**Supporting Figure S4_Maida**


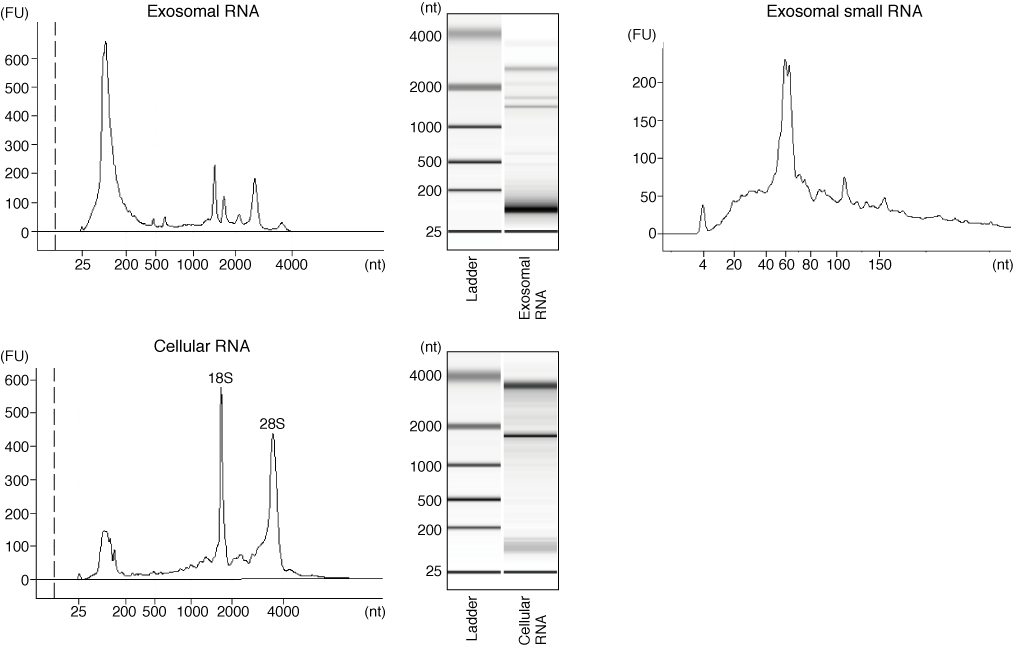


**Supporting Figure S4. Characterization of exosomal RNAs.**

A Bioanalyzer and RNA pico chips (left panels) or small RNA chip (right panel) were used to determine the size distribution of RNAs within each sample of total RNA extracted from Ishikawa cells or Ishikawa-derived exosomes.


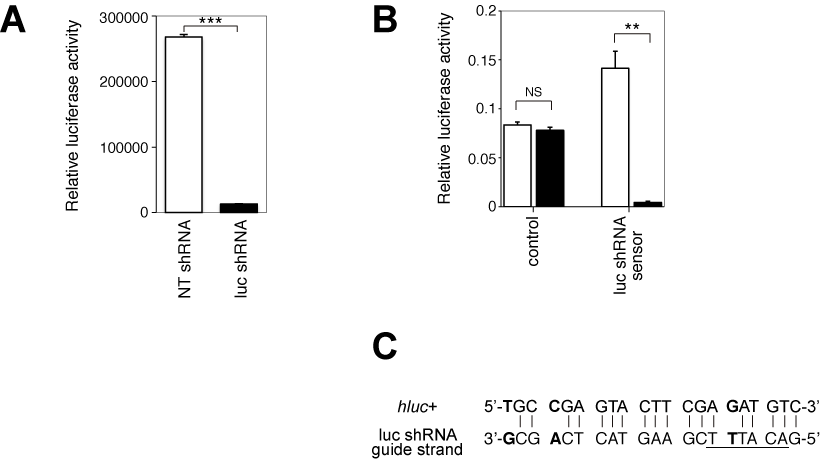
**Supporting Figure S5_Maida**

**Supporting Figure S5. luc shRNA targets perfectly complementary sequences.**

**A.** Adequate silencing of firefly luciferase by luc shRNA. NT shRNA-expressing (white bar) and luc shRNA-expressing (black bar) Ishikawa cells were transfected with pGL3-Control Vector, which contains a modified coding region for firefly luciferase (*luc*+). The nucleotides 159-179 of *luc*+ are perfectly complementary to luc shRNA. Luciferase activity was measured after 48 hours of incubation. **B.** Luciferase assay using psiCHECK-2 vectors. NT shRNA-expressing (white bar) and luc shRNA-expressing (black bar) Ishikawa cells were transfected with psiCHECK-2 Vector (control) or psiCHECK-2 vector with a complementary sequence to luc shRNA at the 3’ UTR of the firefly luciferase (luc shRNA sensor). Luciferase activity was measured after 48 hours of incubation. luc shRNA silenced expression of firefly luciferase with perfectly complementary sequences. **C.** Mismatched sequences at the estimated target region of luc shRNA on psiCHECK-2 Vector. The nucleotides at positions 159-179 of the modified firefly luciferase on psiCHECK-2 Vector (*hluc*+) were compared to the sequence of luc shRNA guide strand. Bold letters indicate mismatches. Seed region of luc shRNA is underlined. (Data from the luciferase assay are shown as mean ± SD. n = 3. **, *P* < 0.01; ***, *P* < 0.001; NS, not significant. two-tailed *t*-test)

**Supporting Figure S6_Maida**


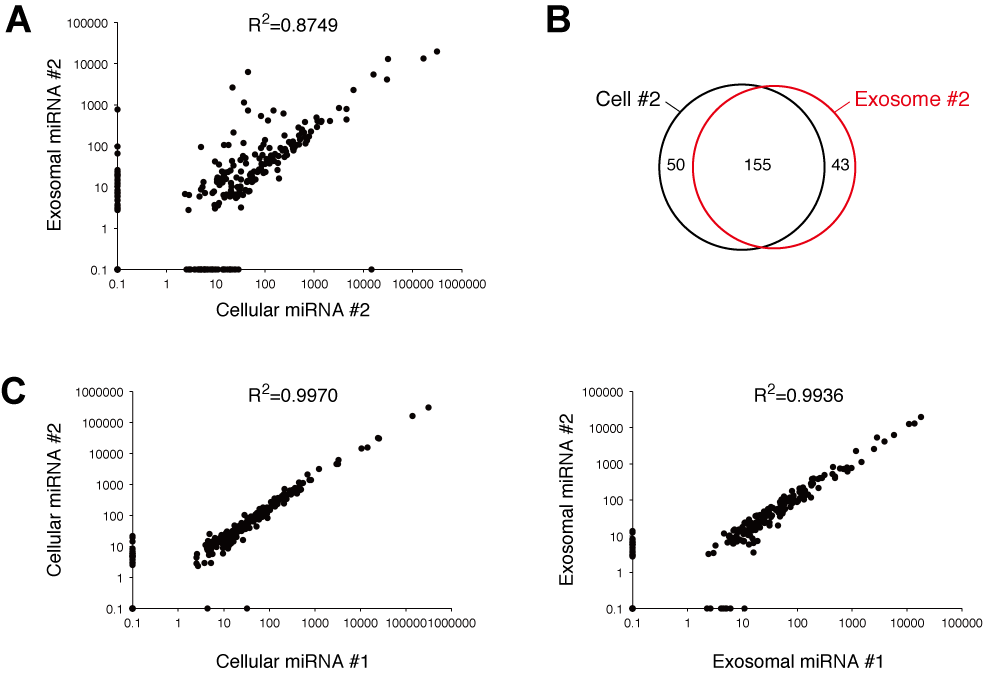


**Supporting Figure S6. Correlation of miRNA expression profiles observed in Ishikawa cells and Ishikawa-derived exosomes.**

miRNA expression profiles of Ishikawa cells and of Ishikawa-derived exosomes were analyzed with miRNA microarrays. **A.** A scatter plot of miRNA expression levels within Ishikawa cells and Ishikawa-derived exosomes for one (#2) of the repeated experiments. **B.** Venn diagram showing the overlap between miRNA signature of Ishikawa cells (Cell #2, black) and that of Ishikawa-derived exosomes (Exosome #2, red) in the experiment #2. **C.** Validation of the repeated experiments. Strong correlation between two independent experiments was ascertained for both cellular miRNAs and exosomal miRNAs. See also Fig. 3.

**Supporting Figure S7_Maida**


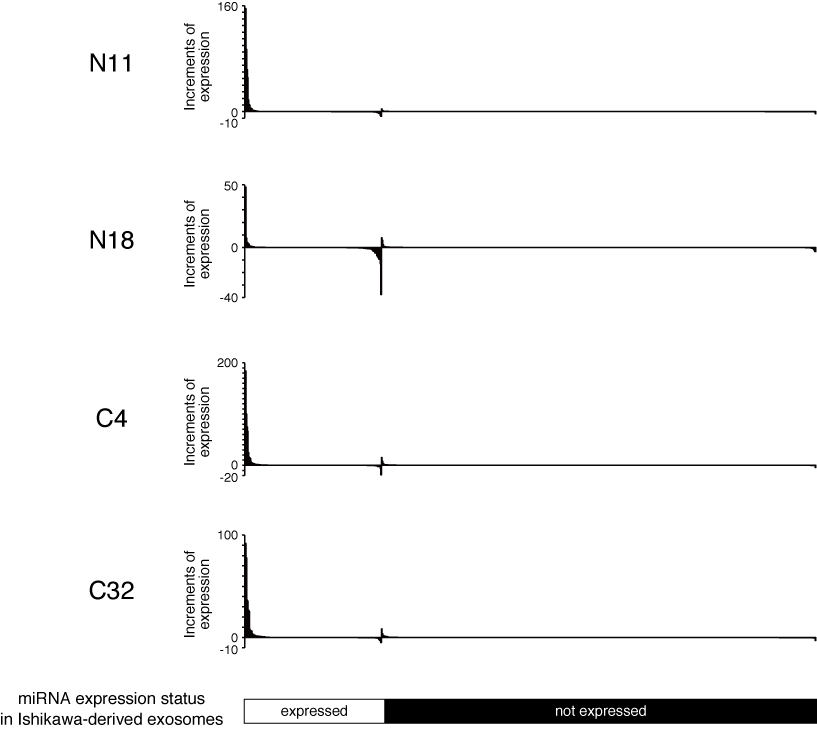


**Supporting Figure S7. Increased expression of the exosomal miRNAs in endometrial fibroblasts treated with Ishikawa-derived exosomes.**

Endometrial fibroblasts were treated with or without Ishikawa-derived exosomes for 5 days. RNAs were then extracted, and miRNA microarray analyses were performed. Increments of miRNA expression upon the exosome treatment were calculated for each miRNA using normalized expression levels by subtracting a normalized intensity observed in cells that were not treated with exosomes from the intensity observed in exosome-treated cells. miRNAs were separated into two groups based on the expression status in Ishikawa-derived exosomes and sorted by increments.
